# Supplementary material for: Insights into the genetic diversity, recombination, and systemic infections with evidence of intracellular maturation of hepadnavirus in cats
Source: PLoS One. 2020 Oct 23;15(10):e0241212. doi: 10.1371/journal.pone.0241212 (PMC7584178; doi:10.1371/journal.pone.0241212)
Supplement: S1 Raw image — (PDF) [file pone.0241212.s001.pdf]

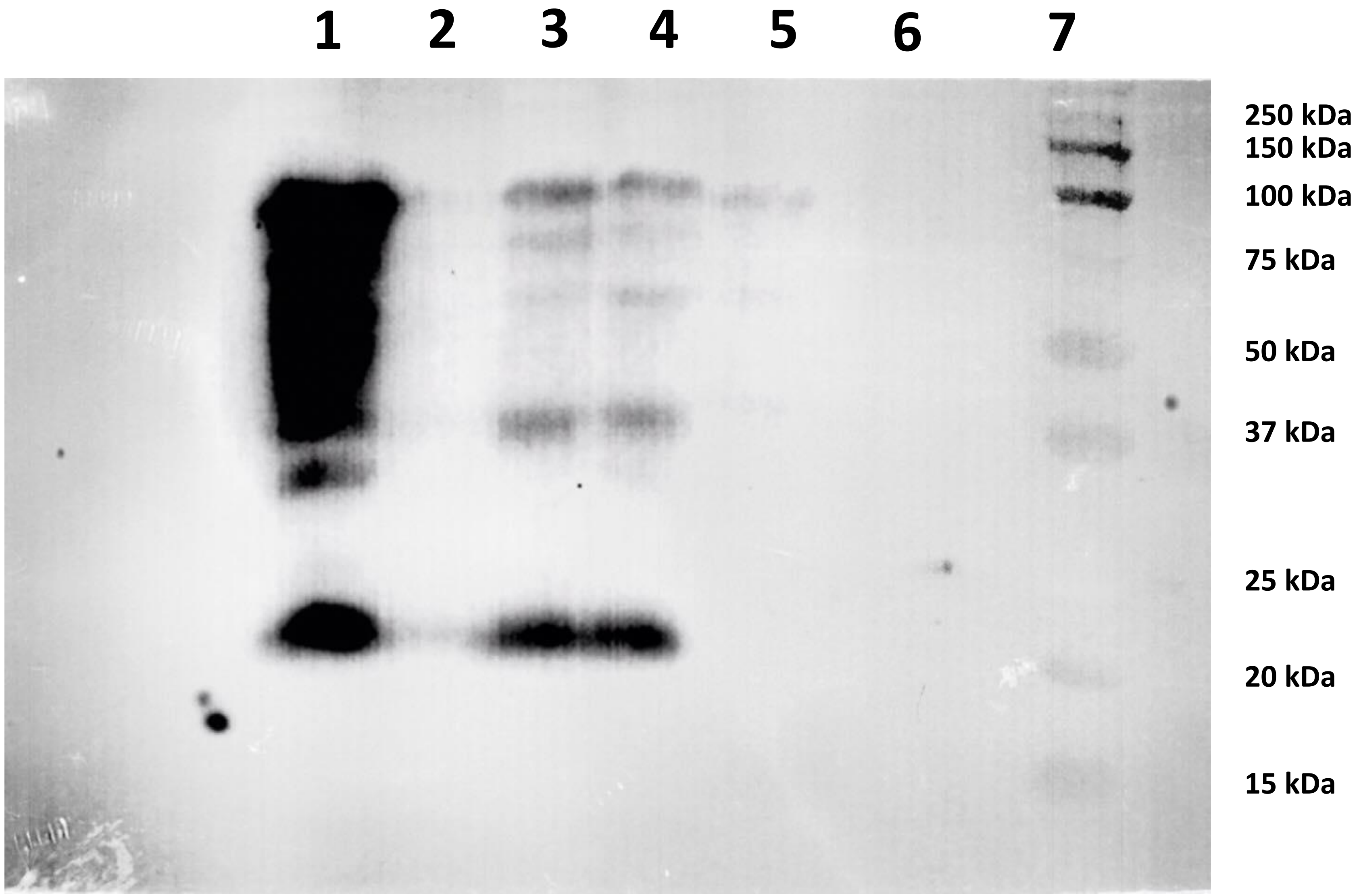

Original western blotting image revealed expected 21 kDa protein band. Lane 1: HBV-infected human liver (158-liver); lane 2: DCH-infected cat liver no. 1 (CPN1-liver); lane 3: DCH-infected cat liver no.2 (CPN2-liver); lane 4: DCH-infected cat liver no. 3 (CPN3-liver); lane 5: DCH-negative cat liver no.1 (045-liver); lane 6: DCH-negative cat liver no. 2 (046-liver); lane 7: size marker indicated protein size. The proteins of interest were visualized with ECL western blotting detection reagents (GE Healthcare) and the western blot imaging was performed using a Pop-Bio Vü Imaging System (Pop-Bio Imaging, Cambridge, UK) according to chemiluminescence detection system.
