# Supplementary material for: Insights into the genetic diversity, recombination, and systemic infections with evidence of intracellular maturation of hepadnavirus in cats
Source: PLoS One. 2020 Oct 23;15(10):e0241212. doi: 10.1371/journal.pone.0241212 (PMC7584178; doi:10.1371/journal.pone.0241212)
Supplement: S1 Table — The primers were designed based on an alignment of the three DCH genomes that were available in GenBank. (PDF) [file pone.0241212.s002.pdf]

Insights into the genetic diversity, recombination, and systemic infections with evidence of intracellular maturation of hepadnavirus in cats

Chutchai Piewbang[1,2], Sabrina Wahyu Wardhani[2,3], Surangkanang Chaiyasak[4], Jakarwan Yostawonkul[3,5], Poowadon Chai-in[5], Suwimon Boonrungsiman[5], Tanit Kasantikul[6], Somporn Techangamsuwan[1,2,*]

S1 Table. The DCH-specific primers used for the full-length genome characterization. The primers were designed based on an alignment of the three DCH genomes that were available in GenBank.

Primer name	Sequence (5'-3')
**DCH_454F**	ATCCCGTCATCATGGGCTT
**DCH_1284**R	GGACGTAGACGAAGGACACGT
**DCH _2113F[a]**	TTGGCACCTGGATTCGCA
**DCH_605R**[a]	AGATGTTCCACACTCTTAGCC
**DCH_1205F**	CCATCGATTTACACACTTCCCA
**DCH_2175R**[a]	ATAACCGTATGCTCCGGAAG
**DCH_2180F[a]**	GTGCTCTGATAACCGTATGCTC
**DCH_1926R**[a]	CTAGAATGGCTACATGGGGTTAG
**DCH_1205F***	CCATCGATTTACACACTTCCCA
**DCH_2180R***	GTGCTCTGATAACCGTATGCTC

* Primer used for extending and re-amplifying the potential recombination break points

[a] Primers derived from Aghazadeh et al. (6)
