# Supplementary material for: Insights into the genetic diversity, recombination, and systemic infections with evidence of intracellular maturation of hepadnavirus in cats
Source: PLoS One. 2020 Oct 23;15(10):e0241212. doi: 10.1371/journal.pone.0241212 (PMC7584178; doi:10.1371/journal.pone.0241212)
Supplement: S1 File — The PCR amplifications were performed using the primers described in S1 Table. (PDF) [file pone.0241212.s003.pdf]

Insights into the genetic diversity, recombination, and systemic infections with evidence of intracellular maturation of hepadnavirus in cats

Chutchai Piewbang[1,2], Sabrina Wahyu Wardhani[2,3], Surangkanang Chaiyasak[4], Jakarwan Yostawonkul[3,5], Poowadon Chai-in[5], Suwimon Boonrungsiman[5], Tanit Kasantikul[6], Somporn Techangamsuwan[1,2,*]

S1 File. Amplification protocols used for characterization of the full-length DCH genome.

The PCR amplifications were performed using the primers described in S1Table with 5 µL of extracted DNA and 10 µM final concentration of each primer in a final reaction volume of 50 µL of Qiagen OneStep RT master mix (Qiagen, Hilden, Germany), as per the manufacturer's protocol. The thermal cycling was performed at 50 °C for 30 min, 95 °C for 5 min, followed by 40 cycles of 95 °C for 1 min, 55 °C for 2 s, and 72 °C for 2 min, and then a final 72 °C for 10 min.
