# Supplementary material for: Insights into the genetic diversity, recombination, and systemic infections with evidence of intracellular maturation of hepadnavirus in cats
Source: PLoS One. 2020 Oct 23;15(10):e0241212. doi: 10.1371/journal.pone.0241212 (PMC7584178; doi:10.1371/journal.pone.0241212)
Supplement: S2 File — (PDF) [file pone.0241212.s004.pdf]

Insights into the genetic diversity, recombination, and systemic infections with evidence of intracellular maturation of hepadnavirus in cats

Chutchai Piewbang[1,2], Sabrina Wahyu Wardhani[2,3], Surangkanang Chaiyasak[4], Jakarwan Yostawonkul[3,5], Poowadon Chai-in[5], Suwimon Boonrungsiman[5], Tanit Kasantikul[6], Somporn Techangamsuwan[1,2,*]

S2 File. Automated IHC protocol for DCH detection in FFPE tissues of DCH-PCR positive moribund cats

Briefly, IHC analysis was executed on the Leica Microsystems Bond maX System (Leica Microsystems, IL, USA). Slides were initially incubated for 60 min at 60 ^{0}C and then treated with Bond Dewax Solution (Leica Microsystems). Epitope retrieval was performed by incubating the sections in Bond Epitope Retrieval Solution 2 (Leica Microsystems) for 30 min at 100 ^{0}C. The IHC analysis was performed using the Bond Polymer Refine Detection kit (Leica Microsystems), a three-step indirect immune-peroxidase technique. The primary antibody against HBcAg used in the manual IHC was applied for 40 min at room temperature followed by three consecutive rinses with Bond Wash Solution (Leica Microsystems). Then, 3% (v/v) H_2O_2 was applied for 5 min and rinsed off three times with Bond Wash Solution. Next, the Post Primary Polymer (Leica Microsystems) was applied for 10 min, rinsed three times as above before the Polymer Poly-HRP IgG (Leica Microsystems) was applied for 10 min, rinsed three times as above and then once with dH_2O before the DAB chromogen was applied for 5 min, followed by triple dH_2O rinses. Slides were subsequently counterstained with hematoxylin. A HBV-infected human liver section and cat sections incubated with normal rabbit IgG NI01 slide, as used in the manual IHC, served as positive and negative controls, respectively.
