# Supplementary figures and images for: Insights into the genetic diversity, recombination, and systemic infections with evidence of intracellular maturation of hepadnavirus in cats
Source: PLoS One. 2020 Oct 23;15(10):e0241212. doi: 10.1371/journal.pone.0241212 (PMC7584178; doi:10.1371/journal.pone.0241212)

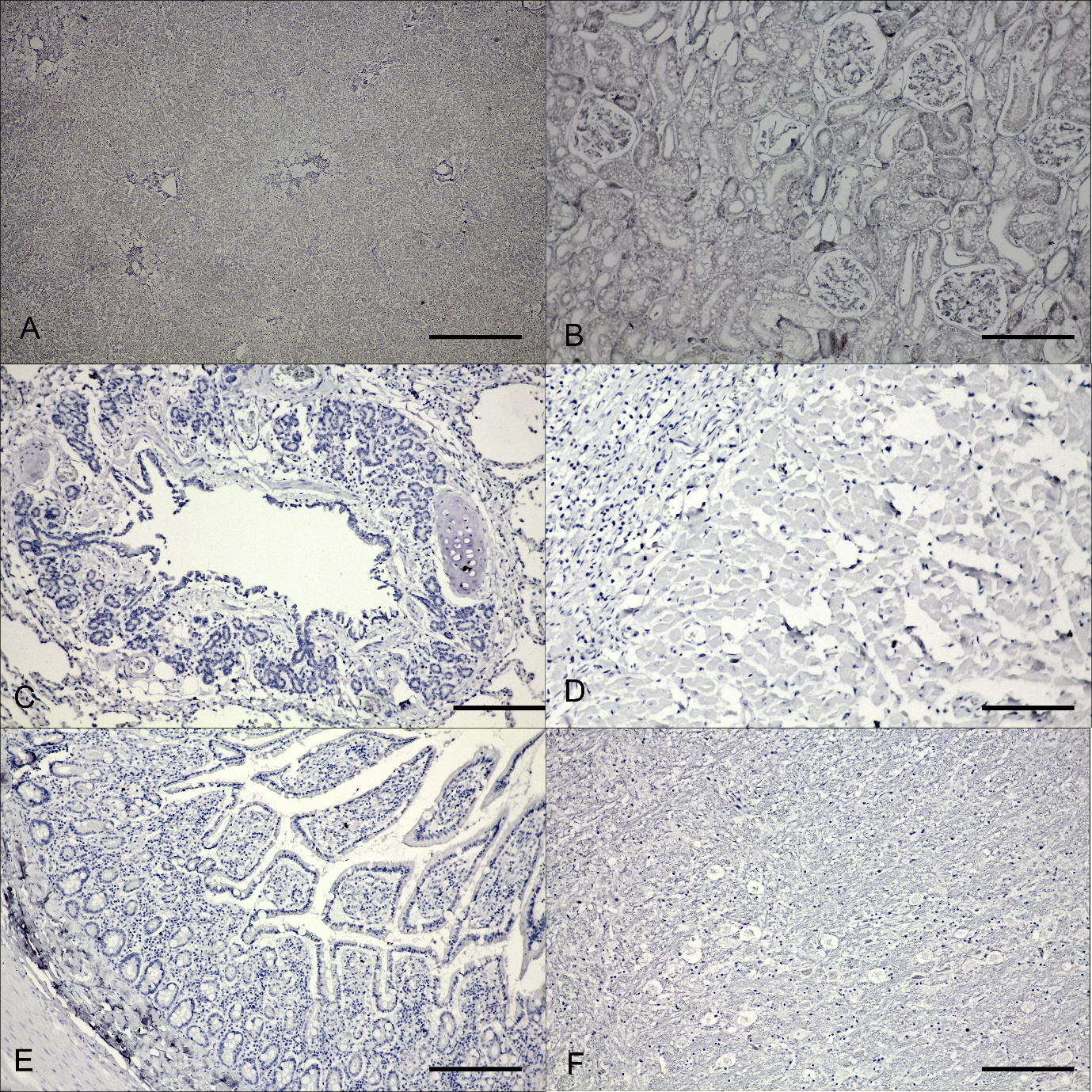

Supplement: S1 Fig — No reaction is present within an incubation of normal rabbit IgG antibody NI01control in the (A) liver, (B) kidney, (C) lung, and (D) heart sections of cat no. 3, the (E) intestine of cat no. 2, and (F) brain of cat no. 1. (TIF) [file pone.0241212.s005.tif]

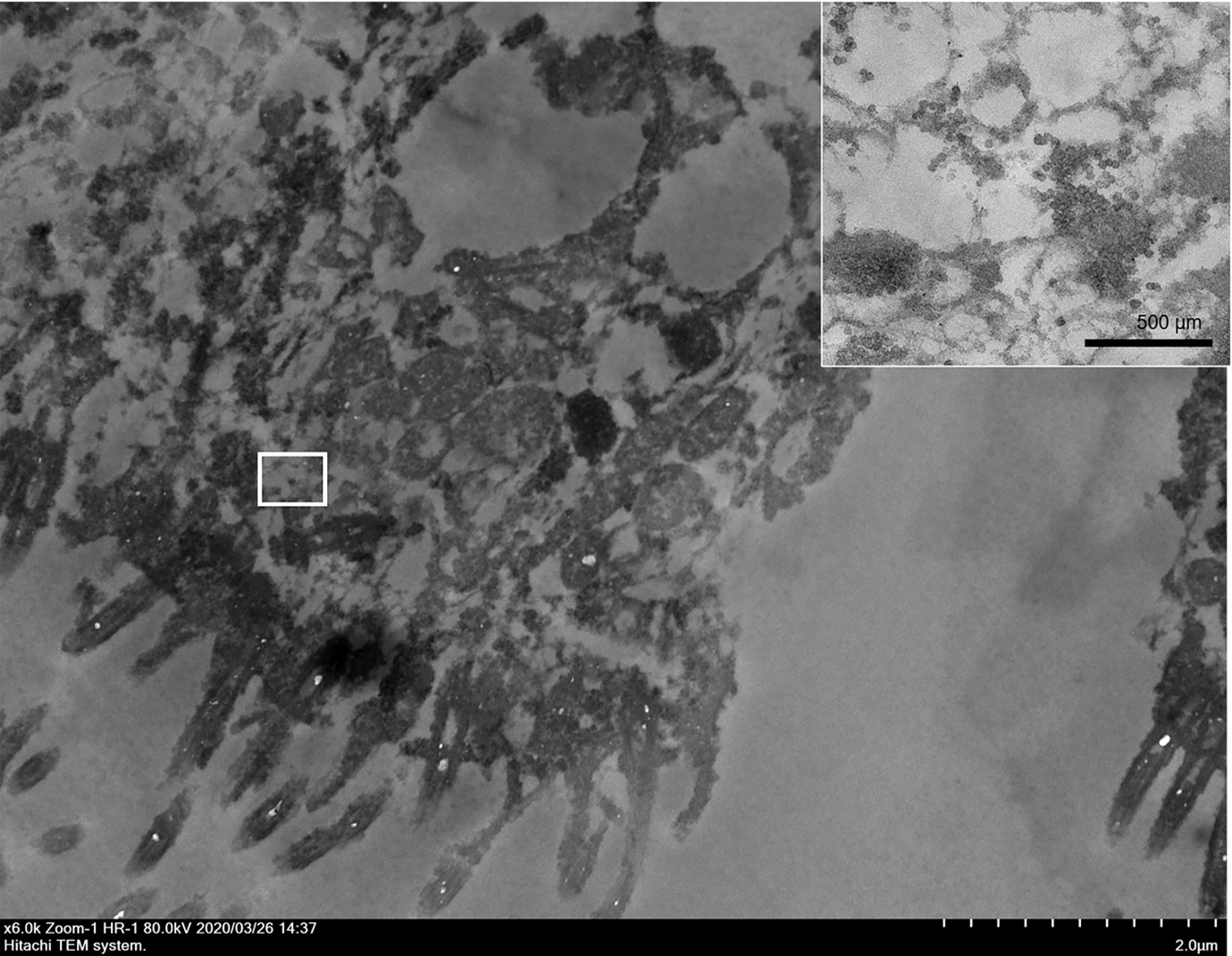

Supplement: S2 Fig — Representative TEM image showing the ultrastructure of the bronchial epithelium and the diffuse electron-dense particles in the cytoplasm (inset). Bar indicates as in figure. (TIF) [file pone.0241212.s006.tif]

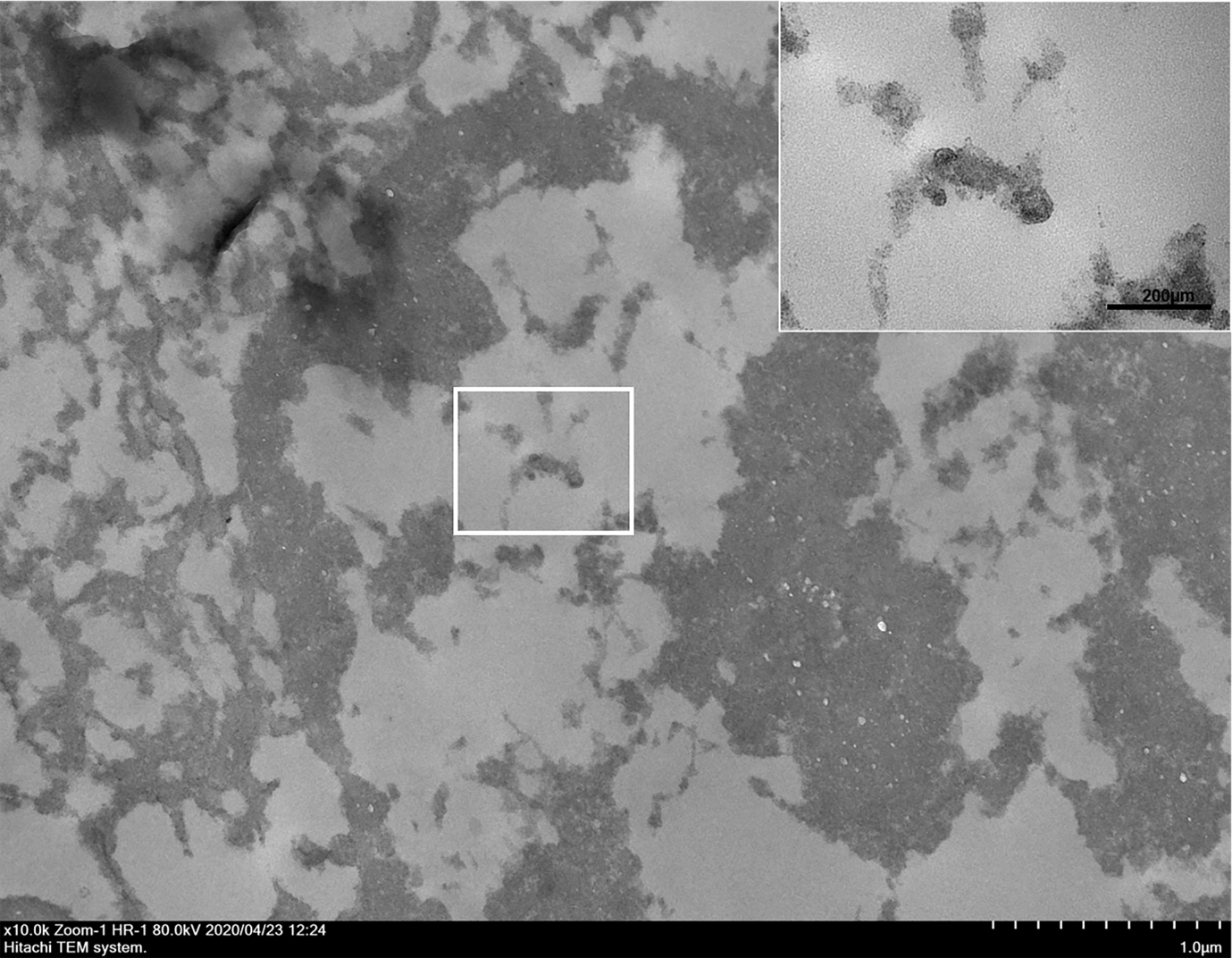

Supplement: S3 Fig — Representative TEM image showing the ultrastructure of the bronchial glandular epithelium and the focal cluster of electron-dense particles in the nucleus (inset). Bar indicates as in figure. (TIF) [file pone.0241212.s007.tif]
